# Focal Hepatic Steatosis Mimicking a Hilar Tumor With Rapid Resolution After Glycemic Control: A Case Report

**DOI:** 10.7759/cureus.102666

**Published:** 2026-01-30

**Authors:** Hiroteru Kamimura, Hiroshi Suzuki, Azusa Sone, Makoto Miyazawa, Yuzo Kawata, Yuko Komoro, Yosuke Horii, Takanori Tsujimura, Hirohito Sone, Shuji Terai

**Affiliations:** 1 Gastroenterology and Hepatology, Graduate School of Medical and Dental Sciences, Niigata University, Niigata, JPN; 2 Hematology, Endocrinology, and Metabolism, Graduate School of Medical and Dental Sciences, Niigata University, Niigata, JPN; 3 Nutrition Management, Nutrition Support Team (NST), Niigata University Medical and Dental Hospital, Niigata, JPN; 4 Pharmacy, Niigata University Medical and Dental Hospital, Niigata, JPN; 5 Radiology and Radiation Oncology, Graduate School of Medical and Dental Sciences, Niigata University, Niigata, JPN; 6 Dysphagia Rehabilitation, Graduate School of Medical and Dental Sciences, Niigata University, Niigata, JPN

**Keywords:** chemical shift mri, diabetes mellitus, focal hepatic steatosis, perihilar cholangiocarcinoma, pseudolesion, segment iv

## Abstract

Focal hepatic steatosis can present as a mass-like pseudolesion, particularly in segment IV (S4) adjacent to the hepatic hilum, and may raise concern for perihilar malignancy. We report a 56-year-old woman with severe hyperglycemia (random plasma glucose 564 mg/dL; HbA1c 12.4%) in whom ultrasonography and computed tomography revealed a small hilar S4 lesion suspicious for a perihilar tumor. Magnetic resonance imaging demonstrated a prominent signal drop on opposed-phase chemical shift imaging without diffusion restriction and without biliary ductal abnormality on MR cholangiopancreatography, favoring focal steatosis rather than neoplasm. Based on these findings, we prioritized metabolic optimization with short-interval follow-up rather than immediate biopsy. After intensive glycemic control, HbA1c improved to 6.6%, and the lesion completely disappeared within three months, confirmed sequentially on ultrasonography, contrast-enhanced CT, and MRI. This case highlights a practical strategy: when chemical shift MRI supports focal steatosis and ancillary malignant features are absent, metabolic optimization followed by short-interval follow-up imaging can confirm the diagnosis and avoid unnecessary invasive procedures.

## Introduction

Metabolically-dysfunction-associated steatotic liver disease is highly prevalent in type 2 diabetes mellitus (T2DM), with an updated meta-analysis reporting a pooled nonalcoholic fatty liver disease (NAFLD) prevalence of 65.0% in T2DM, underscoring how frequently steatosis-related imaging mimics may be encountered in routine practice [1]. Hepatic steatosis is typically diffuse; however, it can present in focal, multifocal/multinodular, or perivascular distributions, and focal fat sparing may also occur, patterns that may simulate a true mass (i.e., “pseudotumor”) on ultrasonography or CT, including hypodense perihilar-appearing lesions [2]. In equivocal cases, MRI, particularly chemical shift imaging, serves as a robust problem-solving modality by confirming intralesional fat and helping avoid unnecessary invasive work-up when malignant features are absent [2].

Among these mimics, segment IV (S4) adjacent to the hepatic hilum is a classic site for “pseudolesions,” largely attributable to regional hemodynamic variation from so-called third inflow (non-portal venous inflow reaching the liver via small aberrant venous drainage pathways beyond the main portal vein) [3-7]. Aberrant gastric venous drainage and pancreaticoduodenal venous routes, as well as the parabiliary venous system (PVS), can deliver insulin- and substrate-rich blood to perihilar S4, creating localized perfusion and metabolic gradients that favor focal fat deposition or focal sparing [3-7]. Consequently, a perihilar S4 pseudolesion may be mistaken for malignancy, most concerningly perihilar cholangiocarcinoma, particularly when it appears as a discrete low-attenuation lesion on CT [3-7].

We describe a patient with severe hyperglycemia who developed a hilar S4 lesion initially concerning for a perihilar tumor. The lesion resolved completely within three months after intensive glycemic control, with time-dependent confirmation on ultrasonography, CT, and MRI. This case highlights a pragmatic diagnostic strategy: metabolic optimization followed by short-interval follow-up imaging when MRI supports focal steatosis and malignant imaging features are absent [2].

## Case presentation

A 56-year-old woman with a history of borderline diabetes and dyslipidemia presented with an acute onset of polydipsia and urinary frequency. She did not drink alcohol and had never smoked. No symptoms specifically attributable to hepatic steatosis were noted; the presenting symptoms were driven by severe hyperglycemia. On presentation, random plasma glucose was 564 mg/dL, and HbA1c was 12.4%. Urinalysis showed no ketonuria. Endogenous insulin secretion was reduced (fasting plasma glucose 180 mg/dL with fasting serum C-peptide 0.94 ng/mL; C-peptide index 0.52). Anti-glutamic acid decarboxylase and anti-IA-2 antibodies were negative. Mild elevation of liver enzymes was present (aspartate aminotransferase (AST) 81 IU/L, alanine aminotransferase (ALT) 48 IU/L), and hepatitis virus markers were negative. Tumor markers were not suggestive of malignancy (carcinoembryonic antigen (CEA) 1.7 ng/mL; carbohydrate antigen 19-9 (CA19-9) 40 U/mL). CA19-9, which was considered clinically nonspecific (potentially related to benign hepatobiliary conditions/steatotic liver change), normalized during follow-up. Selected laboratory data are provided in Table 1.

**Table 1:** Laboratory findings on admission WBC: white blood cell count; RBC: red blood cell count; Plt: platelet count; MCV: mean corpuscular volume; MCH: mean corpuscular hemoglobin; MCHC: mean corpuscular hemoglobin concentration; ALP: alkaline phosphatase; AST: aspartate aminotransferase; ALT: alanine aminotransferase; T-Bil: total bilirubin; D-Bil: direct bilirubin; I-Bil: indirect bilirubin; BUN: blood urea nitrogen; Cr: creatinine; eGFR: estimated glomerular filtration rate; UA: uric acid; AMY: amylase; CK: creatine kinase; Na: sodium; K: potassium; Cl: chloride; Ca: calcium; P: inorganic phosphate; Alb: albumin; TP: total protein; TG: triglycerides; HDL-C: high-density lipoprotein cholesterol; LDL-C: low-density lipoprotein cholesterol; CRP: C-reactive protein; RPG: random plasma glucose; HbA1c: hemoglobin A1c; GA: glycated albumin; CPR: C-peptide; GAD: glutamic acid decarboxylase; TSH: thyroid-stimulating hormone; FT3: free triiodothyronine; FT4: free thyroxine; CEA: carcinoembryonic antigen; CA19-9: carbohydrate antigen 19-9; IFCC: International Federation of Clinical Chemistry and Laboratory Medicine; NGSP: National Glycohemoglobin Standardization Program

Parameter	Patient Value	Reference Range
Complete blood count		
WBC	5,980 /µL	3,300–8,600 /µL
RBC	468 ×10⁴ /µL	376–500 ×10⁴ /µL
Neutrophils	56.70 %	40–70 %
Lymphocytes	39.00 %	20–45 %
Eosinophils	0.70 %	0–6 %
Basophils	0.30 %	0–2 %
Monocytes	3.30 %	2–8 %
Platelets	21.1 ×10⁴ /µL	15.8–34.8 ×10⁴ /µL
MCV	91.2 fL	83–99 fL
MCH	31.4 pg	27–34 pg
MCHC	34.40 %	31–36 %
Biochemistry		
ALP	177 IU/L	38–113 U/L (IFCC, adult)
AST	81 IU/L	13–33 U/L
ALT	48 IU/L	≤30 U/L
T-Bil	0.7 mg/dL	0.4–1.5 mg/dL
D-Bil	0.1 mg/dL	0.0–0.3 mg/dL
I-Bil	0.6 mg/dL	0.2–1.2 mg/dL
BUN	13 mg/dL	8–20 mg/dL
Cr	0.51 mg/dL	0.46–0.79 mg/dL
eGFR	88.1 mL/min	≥60 mL/min/1.73m²
UA	4.1 mg/dL	2.6–7.0 mg/dL
AMY	71 IU/L	44–132 U/L
CK	73 IU/L	41–153 U/L
Na	139 mEq/L	138–145 mEq/L
K	3.8 mEq/L	3.6–4.8 mEq/L
Cl	102 mEq/L	101–108 mEq/L
Ca	9.6 mg/dL	8.8–10.1 mg/dL
P	3.7 mg/dL	2.5–4.5 mg/dL
Alb	4.4 g/dL	4.1–5.1 g/dL
TP	7.0 g/dL	6.6–8.1 g/dL
TG	103 mg/dL	<150 mg/dL
HDL-C	64 mg/dL	≥40 mg/dL
LDL-C	114 mg/dL	<120 mg/dL
CRP	0.05 mg/dL	<0.30 mg/dL
Glucose metabolism/Others		
RPG	564 mg/dL	70–139 mg/dL
HbA1c	12.40 %	4.6–6.2 % (NGSP)
GA	36.20 %	11–16 %
CPR	0.94 ng/mL	0.6–2.0 ng/mL
Anti-GAD Ab	<5.0 U/mL	<5.0 U/mL
Endocrinology		
TSH	1.66 µIU/mL	0.5–5.0 µIU/mL
FT3	2.9 pg/mL	2.3–4.0 pg/mL
FT4	1.30 ng/dL	0.9–1.7 ng/dL
Tumor markers		
CEA	1.7 ng/mL	<5.0 ng/mL
CA19-9	40 U/mL	<37 U/mL
Urinalysis		
Color	Yellow-brown	Straw to yellow
Specific gravity	1.009	1.005–1.030
pH	6	4.5–8.0
Protein	(±)	(−)
Glucose	(+)	(−)
Occult blood	(1+)	(−)
Urobilinogen	Normal	Normal（0.1–1.0 EU/dL）
Bilirubin	(−)	(−)
Ketone bodies	(−)	(−)
Leukocytes	(1+)	(−)
Nitrite	(−)	(−)

**Figure 1:**
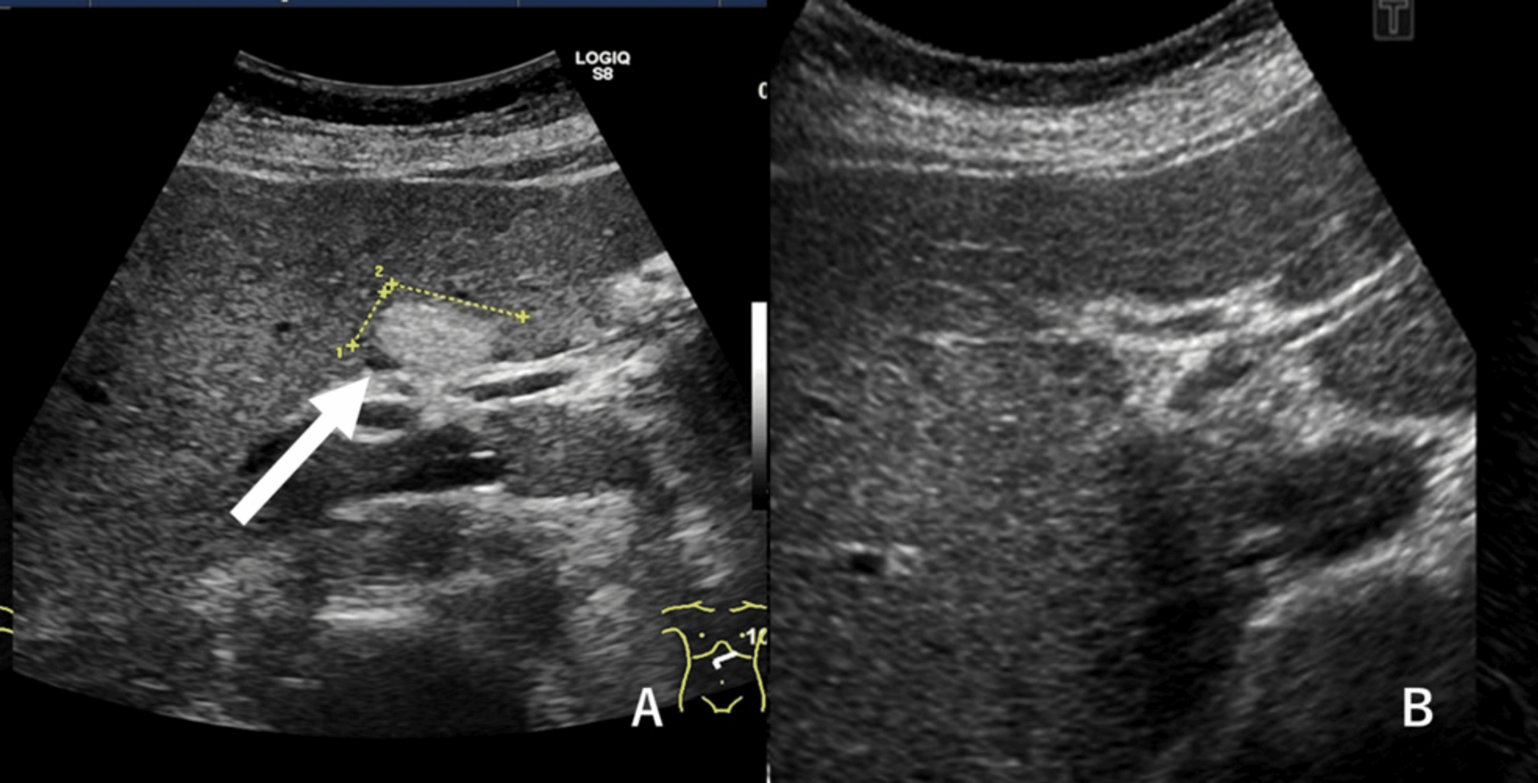
Abdominal ultrasonography findings in segment IV (S4) A: baseline ultrasonography shows a hyperechoic lesion (approximately 25 mm) in segment IV (S4) near the hepatic hilum (arrow); B: follow-up ultrasonography three months after intensive glycemic control shows complete disappearance of the lesion. S4: segment IV; US: ultrasonography

**Figure 2:**
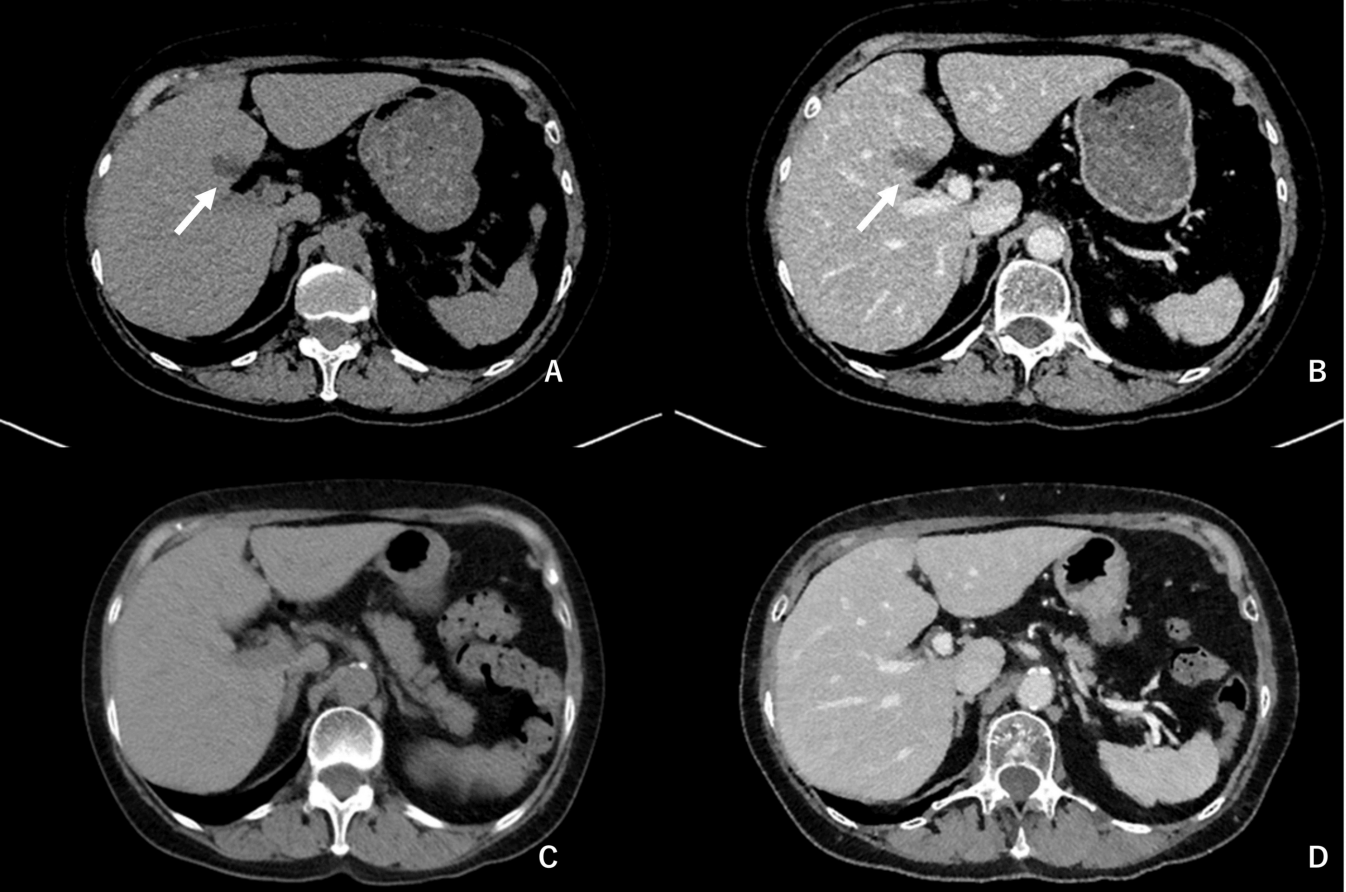
CT findings of the S4 steatotic pseudolesion A: non-contrast CT at presentation shows an oval hypoattenuating lesion (approximately 11 × 23 mm) in segment IV (S4) near the hepatic hilum (arrow); B: contrast-enhanced CT at presentation shows a corresponding hypoenhancing nodule (arrow); C: non-contrast CT three months after treatment initiation shows complete resolution of the lesion; D: contrast-enhanced CT at the same time point confirms disappearance of the lesion. CT: computed tomography; S4: segment IV

Abdominal ultrasonography demonstrated a hyperechoic lesion approximately 25 mm in diameter in S4 near the hepatic hilum (Figure 1A). Non-contrast CT revealed an oval hypoattenuating lesion (approximately 11 × 23 mm) in the same region (Figure 2A), and contrast-enhanced CT demonstrated a corresponding hypoenhancing nodule (Figure 2B). Given its hilar location and hypoenhancement, a perihilar malignant process was considered in the initial differential diagnosis.

**Figure 3:**
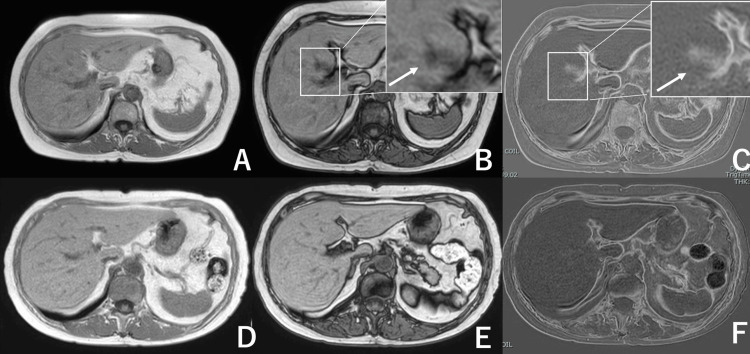
Magnetic resonance imaging (MRI) demonstrates intralesional fat and subsequent complete resolution A: T1-weighted in-phase image shows a focal lesion in segment IV (S4); B: the lesion shows marked signal loss on the opposed-phase image compared with the in-phase image, consistent with intralesional fat (arrow); C: diffusion-weighted imaging shows no diffusion restriction (arrow) (magnified insets are shown in the upper right corners of panels B and C); D–F: follow-up MRI three months after intensive glycemic control shows complete disappearance of the lesion on in-phase and opposed-phase images and no abnormality on diffusion-weighted imaging. DWI: diffusion-weighted imaging; MRI: magnetic resonance imaging; S4: segment IV

MRI with MR cholangiopancreatography demonstrated no bile duct wall thickening or upstream biliary dilatation. Diffusion-weighted imaging showed no diffusion restriction. Chemical shift imaging demonstrated a marked signal drop on opposed-phase images compared with in-phase images, consistent with intralesional fat (Figures 3A-3C). Collectively, these findings favored focal steatosis (pseudolesion) rather than neoplasm.

Because MRI supported intralesional fat and there were no ancillary malignant features (biliary abnormality, diffusion restriction), we prioritized intensive metabolic optimization with short-interval follow-up imaging rather than immediate invasive procedures such as biopsy. 

The patient underwent dietary therapy (1,400 kcal/day; carbohydrate 45% of total energy) and intensive glycemic management concomitantly with basal-bolus insulin (insulin degludec plus insulin lispro; total daily dose approximately 14 units) and sitagliptin (50 mg/day). Glycemic control improved promptly.

Three months after treatment initiation, AST and ALT normalized, and HbA1c decreased to 6.6%. Body weight decreased from 57 kg to 54 kg during follow-up. Follow-up ultrasonography, CT (non-contrast and contrast-enhanced), and MRI demonstrated complete disappearance of the S4 lesion (Figure 1B; Figures 2C-2D; Figures 3D-3F). Given concordant resolution across imaging modalities and the benign MRI signature at baseline, no biopsy or surgery was performed. The patient remained clinically stable with no recurrence during a one-year follow-up period.　

This case report was approved by the Ethics Committee/Institutional Review Board of Niigata University (approval number: 2023-0009). Written informed consent was obtained from the patient for publication of this case report and accompanying images.

## Discussion

This case highlights an S4 focal steatotic pseudolesion adjacent to the hepatic hilum that mimicked a perihilar tumor on initial ultrasonography and CT, yet disappeared completely within three months after intensive glycemic control. The central clinical message is the value of combining MRI confirmation of intralesional fat and short-interval time-dependent imaging to establish benignity, thereby avoiding unnecessary invasive procedures.

Hemodynamic basis of S4 pseudolesions: third inflow and regional gradients

S4 is a characteristic site for focal fatty change and focal fat sparing because regional inflow can be altered by third inflow (non-portal venous inflow via aberrant venous drainage). Prior imaging studies have linked S4 pseudolesions to aberrant gastric venous drainage [3,4], postoperative changes in venous return after gastrectomy [5], parabiliary venous drainage [6], and pancreaticoduodenal venous drainage [7]. These pathways can generate localized perfusion and insulin/substrate gradients that favor focal fat accumulation or focal sparing in the perihilar S4 region. In our patient, the typical hilar S4 location and chemical shift MRI findings were consistent with this mechanism.

Differential diagnosis: distinguishing focal steatosis from perihilar malignancy

The main diagnostic challenge was distinguishing a hilar S4 lesion from perihilar cholangiocarcinoma, metastasis, or inflammatory peribiliary disease. Chemical shift MRI is a key discriminator for focal steatosis, which shows signal drop on opposed-phase images; malignant lesions more commonly show diffusion restriction and other ancillary findings [8,9]. When evaluating suspected perihilar cholangiocarcinoma, careful assessment for bile duct wall thickening, obstruction, and upstream biliary dilatation on MR cholangiopancreatography/CT, alongside diffusion-weighted imaging, is essential [10,11]. In our patient, the absence of biliary abnormality and diffusion restriction, together with marked opposed-phase signal loss, strongly supported focal steatosis. Complete interval resolution across ultrasonography, CT, and MRI within three months further confirmed the diagnosis without the need for biopsy.

Metabolic context and reversibility: glycemic correction can rapidly reduce hepatic fat

Although regional hemodynamics likely underlie S4 pseudolesions, metabolic context remains clinically relevant because hepatic fat content can change dynamically. Contemporary guidelines emphasize the close association between steatotic liver disease and cardiometabolic risk factors, particularly type 2 diabetes, and recommend comprehensive cardiometabolic management in at-risk individuals [12,13]. In this case, rapid metabolic correction temporally paralleled complete radiologic resolution, supporting the concept that hepatic fat can rapidly decrease with improved glycemic control.

Mechanistically, severe hyperglycemia and insulin resistance can increase hepatic fat by boosting de novo lipogenesis and increasing fatty-acid flux from adipose tissue. Intensive insulin-based glycemic control can rapidly suppress lipolysis and reduce substrate oversupply, shifting hepatic lipid balance toward oxidation and very-low-density lipoprotein (VLDL) export; therefore, radiologic improvement over weeks to a few months is plausible. In a third-inflow setting, systemic metabolic normalization may also reduce regional insulin/substrate gradients that favor focal deposition, facilitating rapid resolution.

Interventional data in biopsy-confirmed steatotic liver disease also support metabolic responsiveness: glucagon-like peptide-1 (GLP-1) receptor agonists have shown higher rates of metabolic dysfunction-associated steatohepatitis (MASH) resolution than placebo in phase 2 trials [14,15], and sodium-glucose cotransporter 2 (SGLT2) inhibitors have been reported to reduce liver fat quantified by MRI-proton density fat fraction (MRI-PDFF) in randomized studies and meta-analyses [16,17]. In contrast, evidence for dipeptidyl peptidase-4 (DPP-4) inhibitors is less consistent; sitagliptin was not superior to placebo for reducing MRI-PDFF in a randomized trial [18]. Accordingly, while sitagliptin was included in our regimen, the rapid resolution in this case is most plausibly explained by overall metabolic improvement (dietary therapy and intensive glycemic management) rather than a specific DPP-4 inhibitor effect. Given the rapid glycemic correction, intensive basal-bolus insulin therapy likely played the dominant role, with sitagliptin as adjunctive therapy. The concomitant modest weight loss (57→54 kg) and normalization of mildly elevated CA19-9 further supported a benign, metabolically responsive process.

Clinical implications

When a small, ill-defined lesion is detected in S4 near the hilum, particularly in the setting of marked metabolic derangement, clinicians should consider a third inflow-related pseudolesion. If MRI demonstrates opposed-phase signal loss without diffusion restriction or biliary abnormality, optimizing metabolic control followed by short-interval follow-up imaging can be a rational strategy to confirm the diagnosis and avoid unnecessary invasive procedures.

## Conclusions

A hilar S4 focal steatotic pseudolesion can closely mimic a perihilar tumor on ultrasonography and CT. In this case, chemical shift MRI suggested intralesional fat and showed no diffusion restriction or biliary abnormality. Intensive glycemic management (primarily basal-bolus insulin therapy with dietary therapy and adjunctive sitagliptin) was temporally associated with complete disappearance within three months, confirmed on ultrasonography, CT, and MRI, enabling a confident noninvasive diagnosis. Awareness of this entity and a structured imaging-and-timeline approach can reduce unnecessary invasive procedures. 
